# Comparison of the mucosal and systemic antibody responses in Covid-19 recovered patients with one dose of mRNA vaccine and unexposed subjects with three doses of mRNA vaccines

**DOI:** 10.3389/fimmu.2023.1127401

**Published:** 2023-01-30

**Authors:** Shaojun Liu, Joseph G. S. Tsun, Genevieve P. G. Fung, Grace C. Y. Lui, Kathy Y. Y. Chan, Paul K. S. Chan, Renee W. Y. Chan

**Affiliations:** ^1^ Department of Paediatrics, Faculty of Medicine, The Chinese University of Hong Kong, Hong Kong, Hong Kong SAR, China; ^2^ Department of Medicine and Therapeutics, Faculty of Medicine, The Chinese University of Hong Kong, Hong Kong, Hong Kong SAR, China; ^3^ Department of Microbiology, Faculty of Medicine, The Chinese University of Hong Kong, Hong Kong, Hong Kong SAR, China; ^4^ Hong Kong Hub of Paediatric Excellence, The Chinese University of Hong Kong, Hong Kong, Hong Kong SAR, China; ^5^ Laboratory for Paediatric Respiratory Research, Li Ka Shing Institute of Health Sciences, Faculty of Medicine, The Chinese University of Hong Kong, Hong Kong, Hong Kong SAR, China; ^6^ The Chinese University of Hong Kong (CUHK)- University Medical Center Utrecht (UMCU) Joint Research Laboratory of Respiratory Virus & Immunobiology, Department of Paediatrics, Faculty of Medicine, The Chinese University of Hong Kong, Hong Kong, Hong Kong SAR, China

**Keywords:** mucosal antibody, longitudinal study, mRNA vaccine, hybrid immunity, SARS – CoV – 2

## Abstract

**Background:**

Immunity acquired from natural SARS-CoV-2 infection and vaccine wanes overtime. This longitudinal prospective study compared the effect of a booster vaccine (BNT162b2) in inducing the mucosal (nasal) and serological antibody between Covid-19 recovered patients and healthy unexposed subjects with two dose of mRNA vaccine (vaccine-only group).

**Method:**

Eleven recovered patients and eleven gender-and-age matched unexposed subjects who had mRNA vaccines were recruited. The SARS-CoV-2 spike 1 (S1) protein specific IgA, IgG and the ACE2 binding inhibition to the ancestral SARS-CoV-2 and omicron (BA.1) variant receptor binding domain were measured in their nasal epithelial lining fluid and plasma.

**Result:**

In the recovered group, the booster expanded the nasal IgA dominancy inherited from natural infection to IgA and IgG. They also had a higher S1-specific nasal and plasma IgA and IgG levels with a better inhibition against the omicron BA.1 variant and ancestral SARS-CoV-2 when compared with vaccine-only subjects. The nasal S1-specific IgA induced by natural infection lasted longer than those induced by vaccines while the plasma antibodies of both groups maintained at a high level for at least 21 weeks after booster.

**Conclusion:**

The booster benefited all subjects to obtain neutralizing antibody (NAb) against omicron BA.1 variant in plasma while only the Covid-19 recovered subjects had an extra enrichment in nasal NAb against omicron BA.1 variant.

## 1 Introduction

Coronavirus disease 2019 (Covid-19) is an infectious disease caused by the severe acute respiratory syndrome coronavirus 2 (SARS-CoV-2). We have lived with the SARS-CoV-2 for more than two and a half years while the immune landscape of the population and the SARS-CoV-2 changes over time. Up to now, over 652 million of Covid-19 cases have been reported worldwide and 67.9% of the world population has received at least one dose SARS-CoV-2 vaccine (1). With the introduction of variants of concern (VOC), previously circulating Alpha, Beta, Gamma, Delta, and currently circulating omicron variants, including BA.1, BA.2, BA.3, BA.4, BA.5 and descendent lineages (2), whether a prior infection by certain strain/substrain or the vaccination designed for the original SARS-CoV-2 strain would provide us with sufficient protection against the new VOCs is not guaranteed. Especially, some omicron subvariants, such as BQ.1.1 (a BA.5 subvariant) and XBB (a BA.2 subvariant), are increasing rapidly in several countries, with additional spike mutations that may affect vaccine effectiveness (3). So far, Hong Kong has experienced five waves of Covid-19 outbreaks, with 2.06 million confirmed cases and 10,634 deaths. Since the appearance of the first Covid-19 case in Hong Kong in early 2020, the Department of Health in Hong Kong implemented intense surveillance measures (4) and vigorous contact tracing by the Centre of Health Protection for early quarantine and isolation. It was a very successful strategy to combat the first four waves of Covid-19 between January 2020 and January 2021. The fifth wave caused by the omicron variant, however, resulted in a total of >1 million cases and >9,000 Covid-19 associated deaths from January 6 to Oct 23, 2022 (5).

Airway epithelium is one of the first infected human tissues by SARS-CoV2. Studies have shown that angiotensin converting enzyme-2 (ACE2) (6) and transmembrane proteases serine 2 (TMPRSS2) (7), which are the main entry factors for SARS-CoV-2, can be identified in human epithelial tissues, including nasal epithelium. Not only do nasal epithelial cells serve as the entry site, but nasal mucosa also acts as the first line of defense against the SARS-CoV-2 entry. While the mucus provides the biochemical barrier, the adaptive immunity on the mucosal surface is equally important to limit the invasion of SARS-CoV-2.

Previously, we measured the SARS-CoV-2 spike 1 (S1)- protein specific antibodies in nasal epithelial lining fluid (NELF) in 81 Covid-19 patients from disease onset to six months after discharge (8) and in 83 unexposed Covid-19 vaccine recipients (9). The induction of nasal antibody response is different between natural infection and mRNA vaccine. We found that in these Covid-19 patients who had no pre-existing immunity from vaccination before their infection (recruited in the early phase of the pandemic, June 2020 – January 2021), their nasal antibody was IgA dominant with barely detectable IgG. Moreover, the nasal IgA was induced earlier than their plasma counterpart, and being detected as early as on the fourth day post-diagnosis. In addition, the NELF could inhibit the binding of SARS-CoV-2 to ACE2 which infers its neutralizing ability against SARS-CoV-2 *in vivo*. At six months post-diagnosis, half of recovered subjects still possessed S1-specific IgA in their NELF. In contrast, in the unexposed subjects who received mRNA vaccine, S1-specific IgA and IgG were detectable in 40% and 8% of their NELF by 14 ± 2 days after the first dose and 82% and 68% by 7 ± 2 days after the second dose, respectively. Strikingly, the induction of S1-specific antibody was not detected in the unexposed subjects who took inactivated vaccine.

Currently, the mRNA vaccine is widely used in western countries while the inactivated vaccine is available mainly in developing countries. The enhancement of serological antibody response and cellular immunity could be observed after three, or even four doses of either mRNA vaccine or inactivated vaccine (10, 11). However, most of the studies did not report the local immunological parameters. Concurrently, with the progression of the pandemic as well as the availability of vaccine in different formats, our population has also acquired ‘hybrid’ immunity against SARS-CoV-2 from a combination of scenarios, e.g., a natural infection before the availability of vaccine, vaccination after Covid-19 recovery, unexposed with different vaccine regimens, vaccinated but eventually contracted Covid-19, or any of the above with re-infection. As more individuals were infected with Covid-19, it would be of clinical relevance to evaluate the benefit of further doses of vaccine in enhancing the durability, antibody breadth and the neutralizing potential of mucosal and circulating antibody in subjects after recovery from infection. Moreover, as we found that nasal immunity could be induced by current mRNA vaccine or prior Covid-19 infection, it is important to find out if this could boost the mucosal immune response in the recovered patients.

Unlike the previous VOCs, the omicron variant has thirty-seven mutations in the spike protein, fifteen of which are present in the receptor binding domain (RBD) (12). These mutations enhanced the binding ability to human ACE2 and weakened the binding ability of the antibodies induced by the non-omicron SARS-CoV-2 or vaccine designed against the ancestral strain (13). The reduced neutralizing ability against the omicron variant were observed in serological study (12). However, whether nasal immune response to the omicron variant could be boosted by the current mRNA vaccine (BNT162b2) was not well studied.

This study describes the kinetics of SARS-CoV-2 S1-specific antibody isotypes (IgA and IgG) in the NELF of recovered subjects and vaccine-only subjects from either the day of disease onset or at baseline, i.e., 0-to-2 days before vaccination, to six months of the initial event. The result of this study provides the antibody level, durability and the neutralizing potential in the NELF and plasma against the ancestral and omicron BA.1 strains of SARS-CoV-2. This study will improve our understanding on the antibody isotype kinetics and neutralizing capacity induced among adults with natural infection, vaccination and hybrid immunity.

## 2 Materials and methods

### 2.1 Subject recruitment

The study cohort was followed from the early phase of the pandemic (August to December 2020), before the emergence of SARS-CoV-2 VOCs. Adult patients who were hospitalized with Covid-19 were recruited prospectively if they were within four days of their first RT-PCR-positive result (7). The disease status was confirmed by two RT-PCR tests targeting different regions of the RdRp gene performed by the Public Health Laboratory Service by the Centre of Health Protection. Patients were allocated to the Prince of Wales Hospital in the East New Territories of Hong Kong for clinical management. All patients were unvaccinated and without known prior SARS-CoV-2 infection. Day 0 was considered as the first day of symptoms. Patients were discharged when they were consecutively tested negative for SARS-CoV-2 by RT-PCR or had a viral threshold cycle (CT) value of above 32 and tested positive for nucleocapsid specific serum IgG. All the eleven recovered subjects took one dose of mRNA vaccine (BNT162b2) with at least 180 days interval between infection and vaccination.

Eleven unexposed but vaccinated subjects (control subjects) with similar age (± 3 years old) and same gender were recruited as the vaccine-only group. These subjects were confirmed with no known SARS-CoV-2 infection by CT value of above 40 at 0-2 days before vaccination and the absence of mucosal and serological antibodies against SARS-CoV-2 S1-protein in their baseline specimens. In these vaccine-only subjects, Day 0 was considered as the day of taking the first dose of mRNA vaccine. These subjects took the second dose of mRNA vaccine on Day 21. The third dose (booster dose) of mRNA vaccine was taken at least 180 days after the first dose. They reported that they did not experience any SARS-CoV-2 infection within the study duration.

All research subjects provided written consent for enrollment with approval from the Joint Chinese University of Hong Kong—New Territories East Cluster Clinical Research Ethics Committee (CREC: 2020.076, 2020.4421 and 2021.214).

**Figure 1:**
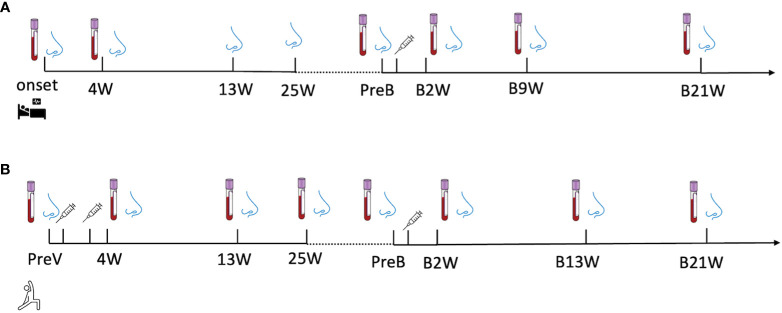
A longitudinal sample collection in **(A)** recovered subjects from the day of diagnosis (disease onset) to twenty-one-week post-vaccination and in **(B)** vaccine-only subjects from 0-to-2 days before the first dose of vaccine to thirteen weeks post-third dose of vaccine. The nose and blood cartoons indicate the time points when NELF and plasma were collected. The syringe cartoon indicates the time points when subjects received vaccines.

Longitudinal biospecimen collections of the recovered group were conducted at eight time points during the in-patient & recovered period and post-vaccination period, including disease onset (onset), 4 weeks (4W), 13 weeks (13W) and 25 weeks (25W) after onset; 0-to-2 days before vaccination (PreB), 2 weeks (B2W), 9 weeks (B9W), 21 weeks (B21W) after vaccination (**Figure 1A**).

The specimens in vaccine-only group were also collected at seven time points, including 0-to-2 days before the first dose (PreV), 4 weeks (4W), 13weeks (13W) and 25weeks (25W) after the first dose of vaccine, 0-to-2 days before the third dose (PreB), 2 weeks (B2W),13 weeks (B13W), 21weeks (B21W) after the third dose (**Figure 1B**).

### 2.2 Severity scoring

Disease severity was categorized as described in the World Health Organization’s Covid-19 clinical management living guidance (14). The disease severity of the symptomatic subjects was categorized into mild (where the clinical symptoms were light, and there was no sign of pneumonia on imaging), moderate (with fever, respiratory tract problems and other symptoms, with imaging suggesting pneumonia), severe (coinciding with any of the following (1): respiratory distress, respiration rate (RR) ≥ 30 times/min (2); oxygen saturation of ≤ 93% in the resting state (3); PaO_2_/FiO_2_ ≤ 300 mmHg (1 mmHg = 0.133 kPa)).

### 2.3 NELF collection

The nasal strip, made of Leukosorb, was inserted into each nostril after 100 µL of sterile saline was instilled followed by a one-minute nose pinch as described (15, 16). All strips were collected and transferred to a sterile collection tube and eluted within 24 h after collection.

### 2.4 Elution of NELF and the preparation of plasma

To elute the NELF, nasal strips were soaked in 300 µL of phosphate-buffered saline (PBS) on ice. The solution and the strips were transferred to a Costar Spin-X (CLS9301) and centrifuged at 4°C. 3 mL of blood was collected by venipuncture and transferred into an EDTA blood tube. Plasma samples were separated by centrifugation at 4°C at 2000 g for 20 min. The specimens were aliquoted into small volume vials and stored at −80°C until the downstream analysis of SARS-CoV-2-specific Ig panels and neutralization tests.

### 2.5 Measurement of SARS-CoV-2 spike protein-specific IgA and IgG

Semi-quantitative measurements of SARS-CoV-2 spike protein (S1 domain)-specific Ig ELISA Kits (Euroimmun, EI 2606-9601 A and EI 2606-9601 G) were used. For this measurement, 1:10 diluted-NELF, as well as 1:100 diluted plasma, were assayed following the manufacturer’s instructions and analyzed with a Synergy HTX Multi-Mode Reader. A semi-quantitative readout was used for the ratio between the sample and the calibrator’s optical density (OD). Data were expressed in the sample/calibrator (S/C) ratio, where a value of ≥ 1.1 was considered positive.

### 2.6 Measurement of SARS-CoV-2 neutralizing antibody against the ancestral SARS-CoV-2 and omicron BA.1

A blocking enzyme-linked immunosorbent assay (GenScript, L00847) was employed as a surrogate of the neutralization test. Briefly, undiluted NELF, 1:10 and 1:100 diluted plasma samples, and controls were processed as per the manufacturer’s instructions. Samples that gave a signal inhibition of ≥ 30% were considered to be SARS-CoV-2 NAb-positive.

### 2.7 Statistical Analysis

The demographic variables of the subjects were described by medians and 95% CI (Confidence Interval) for continuous variables and frequencies and percentages for categorical variables. For the immunoglobulin profile comparisons between the recovered group and the vaccine-only groups were assessed using Wilcoxon matched-pairs signed rank test and Fisher’s exact test, as appropriate. All the S1-specific IgA and IgG levels were expressed as median S/C ratio. All statistical tests were performed using GraphPad version 9.4.1 for the macOS. Differences were considered statistically significant at *p* < 0.05 on a two-tailed test.

## 3 Results

### 3.1 Demographics of the subjects recruited

The cohort consisted of eleven recovered subjects who had participated in an in-patient study (8) and eleven age- and gender-matched seronegative individuals before their first dose of vaccination who had participated in a longitudinal vaccination study since early 2021 (9) (**Table 1**). The median age was 62, ranging from 17-69 years old. Four were male and seven were female. All Covid-19 patients were symptomatic with four mild, four moderate, and three severe cases. The median hospitalization was 14 days, ranging from 9-20 days. The eleven subjects got infected from August 2020 to December 2020. The median duration between onset and one dose of vaccine was 242 days, ranging from 206 days to 311 days. All these eleven subjects took one dose of mRNA vaccine. No death cases were included in this study.

**Table 1:** Demographics of the recovered patients and vaccine-only subjects.

	Recovered Group	Vaccine-only group
Number	11	11
Age (median, range)	62 years old (17–69)	59 years old (20-72)
Gender (male: female)	4:7	
Severity (n)	Mild:4; Moderate:4; Severe:3	Not applicable
Duration of hospitalization (median, range)	14 days (9-20)	
Period of disease onset	August to December 2020	
Duration between onset and vaccination (median, range)	242 days (206-311)	
Period of receiving the 1^st^ dose in vaccine-only subjects	Not applicable	March to July 2021
Duration between 1^st^ & 3^rd^ dose (median, range)		252 days (212-287)

### 3.2 SARS-CoV-2 S1-specific antibody levels in NELF and plasma before the booster dose

**Figure 2:**
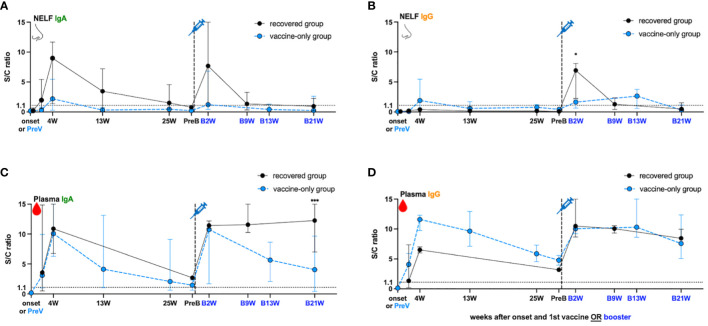
SARS-Cov-2 S1- specific antibody dynamic changes in the **(A, B)** nasal epithelial lining fluid (NELF) and **(C, D)** plasma in recovered group **(black dots)** and vaccine-only group (blue dots). The dynamic changes of S1-specific IgA in NELF **(A)** and plasma **(C)**, S1- specific IgG in NELF **(B)** and plasma **(D)** are measured, respectively. Antibody-level data points above the dotted line (sample/calibrator (S/C) ration ≥ 1.1) are considered positive, while the S/C ratio = 15 indicates the upper detection limit of the assay. In the recovered group, S1-specific Igs are measured at the onset of the disease (onset), 4 weeks (4W), 13 weeks (13W) 25 weeks (25W) weeks after onset, 0 to 2 days before booster (PreB), 2 weeks (B2W), 9 weeks (B9W) and 21 weeks (B21W) after booster dose. In the vaccine-only group, measurement were done on 0 to 2 days before the first dose of vaccine (PreV), 4 weeks (4W), 13 weeks (13W) and 25 weeks (25W) after the first dose, 0 to 2 days before the third dose (PreB), and 2 weeks (B2W), 13 weeks (B13W), 21 weeks (B21W) after the third dose. The median and 95% CI are plotted. The levels of S1-specific Ig were compared between the recovered group and vaccine-only group by the mann-Whitney rank test.

In the recovered group, the NELF collected in the fourth week (4W) of disease onset contained S1-specific IgA (S/C ratio = 8.98, **Figure 2A**) but not IgG (S/C ratio = 0.41, **Figure 2B**), and the IgA declined over the 25 weeks post diagnosis but remained detectable (**Figure 2A**). In contrast, a good induction of S1-specific IgA (S/C ratio = 10.87, **Figure 2C**) and IgG (S/C ratio = 6.50, **Figure 2D**) was seen in the plasma of these patients. Though the circulating IgA and IgG declined over the 25 weeks post diagnosis, they remained detectable at the pre-booster (PreB) time point (**Figures 2C, D**, black dot). The S1-antibody longevity was greater in plasma than it was in the nasal cavity.

In the vaccine-only subjects with two doses of mRNA vaccines, both S1-specific IgA (S/C ratio = 2.2 in NELF, **Figure 2A**; 10.08 in plasma, **Figure 2C**) and IgG (S/C ratio = 1.91 in NELF, **Figure 2B**; 11.59 in plasma, **Figure 2D**) were detected four weeks (4W) after receiving the initial dose. However, the induced S1-specific nasal antibodies declined quickly and became undetectable thirteen weeks post first dose (13W) while the plasma antibodies lasted at least 25 weeks (25W) post first dose and remained detectable at the pre-booster (PreB) time point.

### 3.3 The booster dose of mRNA vaccine induced IgG occurence in the NELF of recovered subjects

While the natural infection did not induce any nasal IgG (**Figure 2B**), one dose of mRNA vaccine could expand the S1-specific immunoglobulin isotype in the NELF with both IgA (S/C ratio = 7.68, **Figure 2A**) and IgG (S/C ratio = 6.92, **Figure 2B**) two weeks after receiving booster (B2W). Nevertheless, both nasal IgA and IgG dropped quickly and became marginally detectable at nine weeks post booster (B9W). In contrast, the circulating IgA and IgG were boosted with a greater magnitude and remained at a high level for at least 21 weeks after the booster dose (B21W). Moreover, the levels of IgG detected at B21W were higher than the convalesce phase (4W) (IgG: S/C ratio = 8.44 at B21W, 6.50 at 4W, *p* = 0.0364, **Figure 2D**), while IgA got a similar trend (S/C ratio = 12.25 at B21W, 10.87 at 4W, *p* = 0.7091, **Figure 2C**).

Unlike the response in the recovered subjects, the booster dose did not induce extensive production of nasal IgA and IgG in the “vaccine-only” subjects who had received two doses of mRNA vaccines. The nasal S1-specific IgA (S/C ratio = 1.23 at B2W, **Figure 2A**) and IgG (S/C ratio = 1.64 at B2W, **Figure 2B**) showed no statistical difference from those induced at 4W. The low level of nasal IgA gradually decreased and became below the positive cut-off in the 13^th^ week of booster (**Figure 2A**, B13W). Nasal IgG lasted subtly longer than IgA and remained positive at B13W. The S/C ratio of specific IgG at B13W showed no significant difference when comparing with that at B2W (S/C ratio = 2.65 B13W vs 1.64 at B2W, *p* = 0.600). In plasma, the third dose induced the production of plasma S1-specific IgA (S/C ratio = 10.75 at B2W, **Figure 2C**) and IgG (S/C ratio = 10.03 at B2W, **Figure 2D**), however, they were not exceeding the highest level found at four weeks after the first dose (4W). Still, both plasma S1-IgA and IgG remained at high levels 13 weeks post third dose, though plasma IgA waned quicker than IgG.

Lastly, we compared the S1-specific antibody levels between recovered subjects and vaccine-only subjects at B2W. We found that the S/C ratio of nasal IgG (6.92 vs 1.64, *p* = 0.0357, **Figure 2B**), and plasma IgA (12.25 vs 4.057, *p* < 0.005 at B21W, **Figure 2C**) in the recovered group were significantly higher than that in the vaccine-only group while nasal IgA and plasma IgG levels showed no significant differences in the two groups (7.68 vs 1.23, *p* = 0.25 at B2W in **Figure 2A**, 10.48 vs 10.29 at B2W in **Figure 2D**).

### 3.4 The plasma of recovered patients exerted a stronger inhibition against the binding of ACE2 to the ancestral SARS-CoV-2 than the vaccine-only group

**Figure 3:**
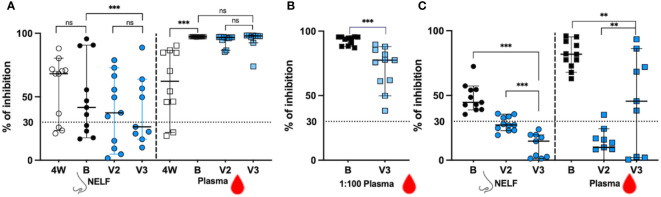
The signal inhibition in the surrogate ACE-2-based neutralization readout. **(A)** The percentage of signal inhibition against ancestral RBD of SARS-CoV-2 by NELF (circle) and plasma (square) of recovered patients (4W: 4weeks after onset; B: 2 weeks post booster) and vaccine-only subjects (V2: 4 weeks post 1^st^ dose; V3: 2 weeks post 3^rd^ dose) are plotted. **(B)** Plasma diluted at 1:100 dilution was used to provide a better resolution to examine the differential neutralization ability between recovered (black square) and control (blue square) subjects. **(C)** The percentage of signal inhibition against SARS-CoV-2 omicron BA.1 by NELF and plasma of recovered patients after booster (B:2 weeks post vaccination) and vaccine-only subjects (V2: 4 weeks post 1^st^ dose; V3:2 weeks post 3^rd^ dose) are plotted. The ≥ 30% signal inhibition cutoff for SARS-CoV-2 NAb detection is interpreted as the sample containing neutralizing antibodies for SARS-CoV-2, indicated by the horizontal dotted line. The median and the 95% CI are shown. Comparison was performed with a Wilcoxon matched-pairs rank test. The asterisks indicate the statistical differences found; **:*p*<0.01. ***:*p*<0.005 and ns: not significant.

In **Figure 3A**, we showed that in the recovered group, 8/11 NELF (open circles) and 9/11 plasma (open squares) samples contained NAb against the ancestral RBD at four weeks after onset (4W). The booster dose did not increase the proportion of positive NELF NAb (7/11, black dots), but enriched all recovered subjects’ plasma NAb (11/11, black squares). In contrast, only 7/11 and 4/9 of the vaccine-only subjects had positive NAb in their NELF after the second dose and after the third dose (blue dots), respectively, while all plasma samples contained NAb against ancestral SARS-CoV-2 RBD at both time points (blue squares). This infers the high potency of mRNA vaccine in inducing circulating NAb.

When the inhibition competence was compared quantitatively before and after booster, no enhancement of NAb against the ancestral SARS-CoV-2 was found in the NELFs of both recovered patients (68.22% at 4W vs 41.65% at B, *p* = 0.3203, **Figure 3A**) and of the vaccine-only group from V2 to V3 (37.4% at V2 and 26.4% at V3, *p* = 0.1289, **Figure 3A**). The booster was only effective in increasing the NAb in the plasma of the recovered group (62.30% at 4W vs 97.31% at B, *p* = 0.0020, **Figure 3A**) while those in the vaccine-only group remained above 96% (96.75% at V2 vs 97.89% at V3, *p* = 0.1289, **Figure 3A**).

When comparing the NAb between the recovered group after booster dose and vaccine-only groups after the third dose, the recovered group had a significantly stronger inhibition effect in their NELF (41.65% vs 26.4%, *p* = 0.0039 in **Figure 3A**). As a high number of plasma samples gave a saturated readout, the plasma samples were diluted in 1:100 for further evaluation. In **Figure 3B**, the 1:100 plasma of the recovered group had a significantly higher percentage of binding inhibition effect than that in the vaccine-only group (94.61% vs 77.79%, *p* = 0.0010).

### 3.5 Booster provided a stronger inhibition against the omicron BA.1 variant in the recovered group

As we did not have enough volume of NELF and plasma sample at four weeks after onset for evaluation of their binding inhibition efficacy towards omicron BA.1 in the recovered group, we could only report the comparison of the inhibition effect between the recovered group after booster dose and vaccine-only group after the third dose, and the inhibition effect between the second dose and third dose within the vaccine-only group (**Figure 3C**). The booster provided all recovered subjects with NAb against the omicron BA.1 in their NELF (44.69%, black dots) and plasma (81.81%, black squares).

Surprisingly, four vaccine-only subjects were found to have positive NELF NAb against omicron BA.1 after the second dose (the median percentage of inhibition = 27.24% at V2, blue circle) while the third dose could not induce detectable NAb against omicron (14.68% at V3, blue circle) which was similar to its effect against ancestral SARS-CoV-2 in the NELF. Intriguingly, all recovered subjects had positive plasma NAb against the omicron BA.1 with median inhibition of 81.81% after the booster dose. The third dose of mRNA vaccine was effective in enhancing the proportion of positive plasma NAb against omicron BA.1 in vaccine-only subjects from 1/11 at V2 to 6/9 at V3. The inhibition efficacy in the plasma of the vaccine-only group rose from below detection limit (9.87%) at V2 to 45.60% at V3 (*p* = 0.0117). Finally, the levels of NAb against omicron BA.1 RBD in the NELF *(p* = 0.0039) and plasma (*p* = 0.0117) of the recovered group were significantly higher than those in the vaccine-only group.

## 4 Discussion

Understanding the mucosal antibody dynamics is an important aspect of evaluating the protection induced by natural infection and any vaccine candidates, as it is one of the keys to sterilizing immunity (17, 18). In animal models, antibodies alone are sufficient to protect against SARS-CoV-2 infection (19). By stimulating mononuclear cells isolated from the tonsil of SARS-CoV-2 infected individuals, Mahallawi et al. confirmed that the SARS-CoV-2 spike primed potent specific memory B cells in nasal associated lymphoid tissue (NALT) (20). The mRNA vaccine, which induces nasal antibody in unexposed subjects, may have the potential to induce the recall of NALT specific memory B cells, thus the increase in nasal Ig levels in the NELF of the recovered subjects receiving their booster. However, no study reported whether the mRNA vaccine alone could induce specific memory B cells in NALT. Tang et al. collected immune cells in bronchoalveolar lavage fluid (BALF) from the Covid-19 infected subjects and vaccine-only subjects. They found that 0.25-8% of total B cells in the infected subjects were RBD^+^ B cells whilst the vaccine-only subjects had a lower percentage at around 0-1% (21). The lack of local RBD^+^ B cells in vaccine-only subjects may explain why the third dose of mRNA vaccine did not boost mucosal Ig extensively in the vaccine-only group. Besides, we noticed the change of nasal antibody isotypes by mRNA vaccine in the recovered groups, with a significant rise of nasal specific IgG while it was negative before the booster. Nasal IgA and IgG induced by the booster lasted only 13 weeks, which is shorter than that acquired after natural infection.

We observed a significant increase of plasma NAb against the RBD of the ancestral strain after one dose of vaccine in the recovered subjects, especially in two of them who had negative NAb before vaccination. The available data in the literature indicated that vaccine-only subjects had weaker neutralizing serum responses, as half of their RBD-specific memory B cells displayed high affinity toward multiple VOCs, while the boosted recovered subjects had their memory B cell pool expanded selectively, matured further and harbored more mutations in their variable V_H_ genes (22). This could explain the boosted neutralizing ability against the ancestral and the omicron BA.1 in the plasma of the recovered group. Interestingly, the nasal neutralizing ability against the ancestral virus induced by mRNA vaccine in the recovered group was much stronger than the vaccine-only group. As the specific memory B cells response in NALT is poorly studied in mRNA vaccinees, no direct evidence illustrates the correlation of memory B cell response and the strong NAb in nasal mucosa.

We found that seven subjects with positive nasal NAb after infection continued to have positive NAb after mRNA vaccine while the NAb in another three subjects remained negative after infection and vaccination. A similar result was observed in the vaccine-only subjects after two and three doses of vaccine (**Figure 3A**). This infers that some subjects might have impaired nasal immune response so that either there were no inductions at the lamina propria, or intrinsic IgA deficiency (23), or the IgA produced did not undergo transcytosis by the polymeric immunoglobulin receptor (pIgR) and therefore, no secretory IgA was detected in the NELF of these subjects (24).

More importantly, all eleven recovered subjects acquired nasal neutralizing antibodies against omicron BA.1 variant after one dose of mRNA vaccine, and their median binding inhibition was significantly stronger than the vaccine-only group. As we lacked nasal NAb data against omicron BA.1 directly after natural infection and there was no published data evaluating the same aspect, it is not clear if the booster was potentiating the inherited effect from the prior natural infection, or it was expanding the antibody breadth. Nevertheless, some studies reported that NAb against the omicron BA.1 variant was detectable but weak in other mucosal fluids, e.g., saliva (25) and BALF (21), after non-omicron Covid-19 infection. In particular, Diem et al. reported that the saliva of the non-infected subjects after three doses of mRNA vaccine had a comparable neutralizing titer against Delta, BA.1, and BA.2 to those recovered subjects (25). In a similar scope, our research suggested that one dose of mRNA vaccine could induce positive nasal NAb in Covid-19 recovered subjects. Together with the better serological Nab, the booster would provide a better immune protection against the omicron variant.

It is noteworthy that three doses of mRNA vaccine did not boost the nasal NAb against the ancestral SARS-CoV-2 nor omicron BA.1 variant in the vaccine-only group, although positive nasal Ig were detected after mRNA vaccine. This could be the reason why mRNA vaccine could not provide sterile protection to its vaccinees. In contrast, a significant increase of plasma NAb against the omicron variant was detected after the third dose, which is consistent with other studies (26, 27) and contributed to its protection against disease severity. These results suggested the intrinsic difference in the induction and potentiation of local and circulating antibody. Currently, different types of intranasal/inhaled vaccines for Covid-19 are under development, including virus-vectored vaccines, protein subunit vaccines, live-attenuated vaccines, and bacterium-vectored vaccines (28). Some of them could produce protection against SARS-CoV-2 in the upper and lower respiratory tract in animal models (29–31). Two virus-vectored vaccines have been approved for use in China (inhaled vaccine produced by CanSino Biologics, Tianjin China), and India (intranasal vaccine produced by Bharat Biotech, Hyderabad India), respectively. Iran has approved one protein subunit intranasal vaccine produced by Razi Vaccine and Serum Institute, for emergency use authorization (EUA) use (32). All in all, the B cell response elicited by the mRNA and other vaccine candidates in NALT and mucosal sites deserve a full examination for better vaccine design.

Apart from humoral immunity, cellular immunity is also critical to combat viral infections. Goel et al. reported that mRNA vaccination generated antigen-specific CD8+ T cells and durable memory CD4+ T cells in SARS-CoV-2 naïve and recovered subjects. They also observed an increasing and fast antibody responses to mRNA vaccine in the short-term without significantly altering antibody decay in the recovered subjects (33). The mucosal-associated T cell response to mRNA vaccine is controversial. One research found that two doses of mRNA vaccine could induce nasal tissue-resident memory (Trm) CD8^+^ T cells in healthy donors, inferring that nasal T cells may be induced and contribute to the protective immunity afforded by this vaccine (34). Another research reported that mRNA vaccine did not elicit strong S-specific CD8^+^ or CD4^+^ T cell responses in the BAL of SARS-CoV-2 naïve subject while BAL from Covid-19 convalescents had higher cytokine-producing CD8^+^ and CD4 T^+^ cells, indicating that mRNA vaccine may offer limited protection against breakthrough infection (21). Further studies in our group would focus on the cellular immune response in these two groups of subjects, especially at the mucosal sites.

Meanwhile, there are several limitations in the current study in terms of the generalizability. First, we had a very small sample size, as research subjects with SARS-CoV-2 exposure before vaccination or vaccination without any SARS-CoV-2 exposure were difficult to recruit. The initial pool of Covid-19 patients during the study period was small due to the unique infectious control measures in Hong Kong. Very soon after the first wave, citizens were provided with Covid-19 vaccine from different vendors, including Comirnaty (mRNA vaccine) and CoronaVac (inactivated vaccine). Therefore, the number of Covid-19 cases without prior vaccination became even less available. Although the number of subjects is small, the overlapping between groups was small with their unique pattern. Therefore, without being able to include more subjects, the current pattern is robust to describe the overall pattern. Second, we did not examine the cellular immunity, e.g., lung-resident memory T cells in these subjects, which is another essential arm of immunity to protect us from the next infection. Third, we only evaluated the IgA and IgG dynamics of the S1-specific antibody but not the antibody against other S ectodomains, e.g., the most potent neutralizers against RBD-2 and the greatest recognition breadth S2-1, and viral proteins. Fourth, due to shortage of sample volume, we were not able to determine if the nasal antibody of the recovered patients exhibited higher cross-neutralization breadth than those induced in unexposed vaccine recipients before their boosters. Lastly, we attempted to recruit patients and unexposed subjects who took inactivated vaccine instead of the mRNA vaccine to provide extra information for patients recovered from Covid-19 to study the response to vaccines with different mechanisms of action. However, we only recruited two within the study period and cannot provide an explicit picture for discussion within this manuscript. Nevertheless, we want to emphasize that mucosal antibody response is an understudied area because of its difficulties in sample collection and standardization for reliable comparisons. The value of our study is obvious because of the eight consecutive longitudinal sample collections together with the long follow up period. Our research provided the dynamics of antibody changes in nasal fluid and plasma with a sampling period covering two years since disease onset.

## 5 Conclusions

In our study, the “hybrid” immune model (infection followed by mRNA vaccine) induced better nasal antibodies, as well as NAb against the ancestral SARS-CoV-2 and omicron BA.1 variant than the vaccine-only subjects. In circulation, both “hybrid” immune model and vaccine-only group demonstrated boosted antibody response. Our findings suggested that one dose of mRNA vaccine is necessary to maintain the plasma NAb against the ancestral SARS-CoV-2 and elicited the NAb against omicron BA.1 variant for recovered subjects during the omicron wave. The third dose would provide extra benefit for people who had no prior SARS-CoV-2 exposure to acquire serological NAb against SARS-CoV-2 VOC, e.g., omicron BA.1. Further studies focusing on the cellular immunity at the mucosal sites will be needed to elucidate the comprehensive outcome of the hybrid immunity. Finally, though the differential pattern between the hybrid and vaccine-only group is robust, cautions should be taken for its generalizability due to its unavoidable small sample size.

## Data availability statement

The original contributions presented in the study are included in the article/supplementary material. Further inquiries can be directed to the corresponding author.

## Ethics statement

The studies involving human participants were reviewed and approved by The Joint Chinese University of Hong Kong—New Territories East Cluster Clinical Research Ethics Committee. The patients/participants provided their written informed consent to participate in this study.

## Author contributions

Conceptualization, formal analysis, SL and RC. Investigation, methodology, SL, JT and RC. Patient recruitment, SL, JT and GL. Resources, GL, PC and RC. Writing—original draft preparation, SL and RC. Writing— SL, JT, GF, GL, KC, PC, RC. Visualization, SL and RC. Supervision, RC. Project administration, SL, JT, RC. Funding acquisition, RC. All authors contributed to the article and approved the submitted version.
